# Mirror Therapy in Patients with Somatoform Pain Disorders—A Pilot Study

**DOI:** 10.3390/bs13050432

**Published:** 2023-05-20

**Authors:** Steffen Philipp Ruf, Larissa Hetterich, Nazar Mazurak, Caroline Rometsch, Anna-Maria Jurjut, Stephan Ott, Anne Herrmann-Werner, Stephan Zipfel, Andreas Stengel

**Affiliations:** 1 Department of Psychosomatic Medicine and Psychotherapy, University Hospital Tübingen, Osianderstr. 5, 72076 Tübingen, Germany; 2 Department of Experimental and Clinical Medicine, University of Florence, Largo Brambilla 3, 50134 Firenze, Italy; 3 Institute of Occupational, Social and Environmental Medicine with Outpatient Clinic, Friedrich-Alexander-Universität Erlangen-Nürnberg, Henkestr. 9-11, 91054 Erlangen, Germany; 4 TIME (Tübingen Institute for Medical Education), Medical Faculty Tübingen, Elfriede-Aulhorn-Str. 10, 72076 Tübingen, Germany; 5 Charité Center for Internal Medicine and Dermatology, Department for Psychosomatic Medicine, Charité-Universitätsmedizin Berlin, Corporate Member of Freie Universität Berlin, Humboldt-Universität zu Berlin, and Berlin Institute of Health, 12203 Berlin, Germany

**Keywords:** chronic pain, mirror therapy, heart rate variability, pain management, pain threshold, pain treatment, psychosomatic disorders, somatoform pain disorders

## Abstract

Patients with chronic pain report reduced quality of life and high symptom burden while often responding insufficiently to treatment options. Mirror therapy has been proven to be effective in treating phantom limb pain and other conditions such as CRPS. This study was designed to investigate the efficacy of mirror therapy in patients with somatoform pain disorders on symptom severity and associated physiological parameters. Fifteen patients with persistent somatoform pain disorder (F45.40) or chronic pain disorder with somatic and psychological factors (F45.41) participated and received four weeks of tablet-based mirror therapy. Symptom severity was measured with established questionnaires, and their thermal detection, pain thresholds, and heart rate variability (HRV) were also assessed. After mirror therapy, pain intensity was reduced (*z* = −2.878, *p* = 0.004), and pain thresholds for cold stimuli were also diminished, i.e., the subjects became more sensitive to cold stimuli (*z* = −2.040, *p* = 0.041). In addition, a reduction of absolute power in the low-frequency band of HRV (*t*(13) = 2.536, *p* = 0.025) was detected. These findings indicate that this intervention may reduce pain intensity and modulate associated physiological parameters. As these results are limited by several factors, e.g., a small sample size and no control group, they should be validated in further studies investigating this novel intervention in these patients.

## 1. Introduction

The prevalence of chronic pain in the general population is estimated to be as high as 40–50 percent [1,2] and poses a significant burden on affected individuals, communities, and healthcare systems in general [3]. As chronic pain can be classified in several ways [4], some conditions involve chronic pain that cannot be adequately explained through structural somatic injury [5]. These somatoform pain disorders share some symptomatic overlap with functional syndromes (e.g., fibromyalgia), as pain is the central component of the presented symptoms. However, they are distinct disorders with major challenges regarding the diagnostics, and treatment as a somatic origin for the maintenance of pain symptoms cannot be found. Additionally, pain symptoms occur in association with psychosocial or emotional problems, which suggests these as the main causative factors [6,7]. The prevalence of somatoform disorders in the general population ranges from one to twenty percent, and, to a large extent, they are chronic somatoform pain disorders [8,9].

In accordance with ICD-10-GM [10], persistent somatoform pain disorder (F45.4) is defined as a severe, tenacious pain that cannot be fully explained by physiological processes, often associated with emotional distress and interpersonal conflicts, and commonly featuring comorbidities, e.g., depression or anxiety disorders [11]. In addition, for the diagnosis of chronic pain disorder with somatic and psychological factors (F45.41), although the origin of the pain may be a physiological process, the current severity of the pain and its maintenance is significantly influenced by psychological factors.

Patients are generally treated using a multimodal treatment approach that can include pharmacological options, psychotherapy, mindfulness-based or biofeedback interventions, and exercise [3,12,13,14,15]. While there are several effective non-pharmacological treatment options for pain disorders, the effect sizes of these interventions for somatoform pain disorders are rather small [16]. Overall, patients are left with treatment options that are not yet fully adequate [3]. As there are lots of patients affected by these disorders [3,6], and they experience a huge limitation in quality of life, including an increased risk of sick leave and work disability [17], new treatment options are therefore warranted.

Mirror therapy is an interventional treatment that was initially intended for patients with phantom limb pain [18,19] but is also used in patients with stroke residuals or complex regional pain syndrome (CRPS) [20,21]. It is mainly performed with a mirror placed in the sagittal plane, dividing the body core while hiding the painful or dysfunctional body area. The healthy limb is visible in the mirror and projected towards the painful side [22]. Patients perform diverse exercises while looking at the healthy limb and its reflection in the mirror, creating the illusion that the painful limb is performing the movement. While the exact underlying mode of action of mirror therapy is still not entirely clear, it is thought to have a therapeutic effect on chronic pain by reversing maladaptive cortical reorganization and increasing cortical excitability by integrating the perception and action of motor movements—the patient observes that limb movement is possible without pain and the visual input can “override” the painful input of the affected limb. This can lead to improvements in functional rehabilitation by facilitating and reactivating motor and sensory pathways in the impaired and unimpaired limb. Mirror therapy may also restore the congruency of faulty sensory and motor output by facilitating descending inhibitory mechanisms, which then result in pain reduction [23,24]. While chronic pain disorders and somatoform pain disorders in particular share some overlap but are clearly distinct from the disorders where mirror therapy has been proven to be efficacious, the underlying physiological rationale [25] could still apply. For example, although patients with chronic somatoform pain disorders do not suffer from phantom pain, their pain conditions may also improve via the restoration of congruence between sensory input and motor output, as well as the modification of learned dysfunctional use of impaired and painful limbs. Mirror therapy may also change the dysfunctional expectations of body movements and activities or reduce pain by promoting descending inhibitory pathways [18,26,27].

To the best of our knowledge, there has been no investigation into the efficacy of mirror therapy for patients with chronic somatoform pain disorders. As chronic pain disorders are often accompanied by distinct autonomous and physiological differences, such as abnormal autonomous functioning [28] and altered pain sensitivity [5], measures to assess potential changes in these domains were also included in this study. For this purpose, the assessment of heart rate variability (HRV) and pain thresholds were chosen, as these are feasible and accessible methods allowing for an efficient and simple evaluation of these autonomous and physiological differences in chronic pain disorders.

The analysis of HRV is a powerful tool for investigating the central regulation of autonomic activity via the sympathetic (SNS) and parasympathetic (PNS) nervous systems [29]. It allows for a non-invasive evaluation of the adaption capabilities of the cardiovascular system, physiological functioning, and its relationship to various psychological phenomena and mechanisms [29,30]. Specifically, parasympathetic activation seems to be decreased in patients with chronic pain [31]. Altered HRV with increased low-frequency and reduced high-frequency power has been found in various pain-related disorders [32,33], indicating an increased sympathetic and reduced vagal tone [34]. Research also suggests sympathetic over-activation and reduced HRV in psychosomatic inpatients [35] and patients with somatic symptom disorder, respectively [33].

Increased sensitivity to painful stimuli has been found in several chronic pain conditions [36,37], and pain sensitivity seems to be negatively correlated with pain thresholds as well [38]. As increased pain thresholds and tolerances may also be relevant in terms of predicting the response to pain treatments [39,40], we also included measurements of pain thresholds in addition to the assessment of HRV.

The study was designed to provide preliminary evidence of the potential efficacy of mirror therapy in a clinical sample of patients with chronic somatoform pain disorders. First, this interventional study aimed to experimentally measure the change in pain intensity before and after using mirror therapy. To find out whether potential improvements were not only reflected in the changes in the self-reported pain intensity but also in more objective parameters, such as HRV and thermal pain thresholds, in which these patients presented distinct alterations, we secondly assessed pain sensitivity to painful stimuli before and after using mirror therapy. We, therefore, expected that if changes in the thermal thresholds occurred, the direction of the effect would be towards a reduced sensitivity to painful stimuli. Third, we examined if possible changes in the self-reported pain intensity were also reflected in alterations of the objective physiological measure of HRV. Here, our hypothesis was that if the pain symptoms were reduced by the intervention, the high-frequency and low-frequency HRV would increase and decrease, respectively.

## 2. Materials and Methods

### 2.1. Sample Description

Patients were consecutively recruited from 2019 to 2021 at the Outpatient Clinic for Psychosomatic Medicine at the University Hospital Tübingen. Fifteen (*n* = 15) patients with persistent somatoform pain disorder (ICD-10-GM: F45.40) or chronic pain disorder with somatic and psychological factors (F45.41) were enrolled in this study. All patients who presented at the Outpatient Clinic to the attending physician and had the necessary diagnosis were contacted individually by the study personnel and evaluated for their suitability to participate in the study. Participating patients were diagnosed by the attending physician according to ICD-10-GM and continued their respective individual multimodal treatment during their participation in the study. To reduce the variability in the data of pain symptoms and physiological parameters due to individual patient treatments (e.g., physiotherapy, pharmacotherapy, or psychotherapy), these were not allowed to change during participation in the study, and no new therapeutic treatment was allowed to be started.

To be included in the study, patients had to be between the ages of 18 and 65, had to be diagnosed with either F45.40 or F45.41, and pain needed to be lateralized, i.e., predominant in the left or right side of the body. By design, mirror therapy requires one functional or pain-free limb to be utilized in the respective exercises, and the pain complaints of patients, therefore, needed to be lateralized in order for the mechanism of mirror therapy to take effect. Patients were excluded from partaking in the study if any of the following applied: recent oncological or documented diagnosis of a psychotic disorder, pregnancy or breastfeeding period, substance abuse, participation in drug trials three months prior, intake of α-blockers, β-blockers, or amitriptyline (dose > 50 mg). Patients with the mentioned medications were excluded due to their effect on the autonomic nervous system. While other medications may also have an effect on cardiac functioning, we focused on the medications that have a clear adrenergic effect, for example, beta-antagonists show definite altering effects on HRV metrics [41].

### 2.2. Study Procedure

All measurements of this study were performed in the autonomous function laboratory of the Department of Psychosomatic Medicine at the University Hospital Tübingen. Baseline characteristics and demographics of the study sample were assessed at the beginning of mirror therapy (T0) digitally via a tablet. At the beginning of this first measurement session, the patients were informed about the study and its design and, afterwards, gave their informed consent for participation. Psychometric (questionnaires), physiological (HRV), and psychophysical (thermal detection and pain thresholds) measurements were taken before (T0) and after (T1) the execution of the mirror therapy program. HRV measurements were obtained before measuring thermal detection and pain thresholds. At the end of the first measurement session, the patients were introduced to and familiarized with the mirror therapy program. During the four-week interval (between T0 and T1), the patients performed guided mirror therapy using a tablet at home for a duration of 15 min each day. All psychometric parameters (e.g., symptom severity, psychological and social functioning) were assessed digitally with a tablet.

### 2.3. Mirror Therapy

In the first sessions, mirror therapy and its implementation were introduced and explained to all subjects by qualified study personnel. The therapy regimen consisted of separate exercises for the upper and lower limbs of the pain-dominant side of the body and were executed by the patients at home. Mirror therapy has been shown to be effective not only when performed in a clinic but also in a home-based setting, with patients showing high adherence to the regimen [19,42,43]. To ensure better comparability regarding the implementation of the therapy and its effects, all patients carried out exercises for the upper and lower limbs, regardless of which individual limb was affected. All exercises were guided via the tablet application Routine Reha (Routine Health GmbH, Düsseldorf, Germany). The Routine Reha application, in total, includes even more versatile options for the teletreatment of patients with phantom limb pain [44]. In our study, however, the app was used exclusively to perform the mirror therapy exercises.

**Figure 1:**
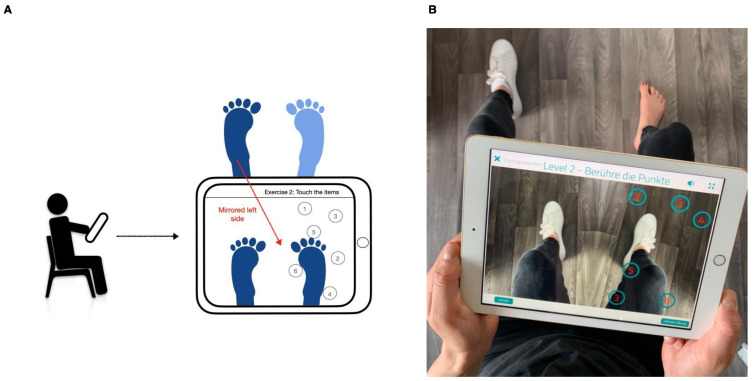
Illustrations of mirror therapy exercises performed by the patients. In this example, pain was localized in the right lower limb. Exercises were therefore performed with the healthy left lower limb, which was mirrored onto the right side. (**A**) Schematic depiction of the exercise; (**B**) realistic representation of the exercise.

The exercises consisted mainly of fine motor tasks in which the extremities had to be moved precisely and unerringly. For example, number points seen in the mirror had to be traced in the correct order, the limbs had to be moved sensibly with or against the clock hand, or certain figures had to be traced. Lower limb exercises were carried out with the tablet utilizing its onboard camera system to reflect the patients’ own movements as conceptualized by a mirror (Figure 1). Exercises for the upper limb used the tablet only as an instructional device and needed a separate table mirror for execution. Subjects were instructed to perform both types of exercise at home for fifteen minutes per day for a total time period of four weeks, with the fifteen minutes divided evenly among three exercises of increasing difficulty for both the upper and lower limbs.

### 2.4. Psychometric Parameters

The psychometric data collection was based on validated questionnaires and consisted of the German Pain Questionnaire (DSF), which is routinely used in clinical applications.

The DSF of the German pain association contains a detailed acquisition of subjective pain symptoms, severity, and associated restriction [45]. In addition, it contains established questionnaires for measuring habitual wellbeing (Marburg Questionnaire on Habitual Well-Being, MFHW, [46]) and assessing depression-, anxiety- and stress-related symptoms (Depression–Anxiety–Stress Scales, DASS [47]). The MFHW consists of Likert-scale questions and addresses the positive abilities of the patients, such as feeling comfortable or fulfilled and satisfaction with work and physical performance. The DSF is a well-established questionnaire routinely used in clinical settings and offers high reliability and content validity [48]. The relevant parameters of the DSF were the score of pain intensity (with scores ranging from 0 to 100), the disability score (ranging from 0 to 6), the score of wellbeing (ranging from 0 to 35), and the scales of the DASS, with scores ranging from 0 to 21. The overall score of pain intensity comprises three numerical rating scale (NRS) questions measuring the *current pain intensity* and *average* as well as the *greatest pain intensity* during the last four weeks. With the exception of the wellbeing score, the following applies: the higher the corresponding values of the DSF scores, the higher the degree of impairment. High scores on the scale of wellbeing indicate higher functioning, i.e., habitual wellbeing.

### 2.5. Physiological Parameters

Heart rate variability (HRV) was recorded before testing the thermal detection and pain thresholds with eMotion Faros 180° (Mega Electronics Ltd., Kuopio, Finland). HRV data analysis was performed with Kubios HRV Premium (ver. 3.3.1, Kubios Ltd., Kuopio, Finland). HRV was assessed during a five-minute sitting resting period in which participants were verbally instructed to relax and not talk or move (as described in [49]). The data were collected with a sampling rate of 1000 Hz and saved on the device for offline analysis. For artifact correction, the automatic correction algorithm of Kubios software was used, which was shown to reliably correct for artifacts in recorded HRV data with high sensitivity and specificity [50]. The parameters of interest were the root mean square of successive differences (RMSSD) as a primary time-domain index for measuring overall short-term HRV as well as frequency-domain indices, i.e., the low-frequency (LF) band (0.04–0.15 Hz) and high-frequency (HF) band (0.15–0.40), reflecting SNS and PNS influences via baroreceptor activity and parasympathetic activity, respectively [51,52].

### 2.6. Psychophysical Parameters

Thermal detection and pain thresholds were measured separately for cold and warm/hot stimuli with the Thermal Sensory Analyzer (TSA-II, Medoc Ltd., Ramat Yishai, Israel), overall resulting in four separate measurements. Using a thermode (3 × 3 cm) on the measured center of the volar forearms, the patients received thermal stimuli with an initial temperature of 32 °C. To avoid testing both cold and hot stimuli on the same area, we applied cold stimuli to the left and warm/hot stimuli to the right forearm. No patient presented with pain in the forearm region. The temperature was increased or decreased by steps of 1 °C/sec (according to the testing protocol in [53]). The stimuli were halted and reset to the starting temperature immediately as soon as the patients indicated that they reached their respective thresholds. The patients were instructed to press a button when sensing cooling or heating of the thermode measuring the cold detection thresholds (CDT) and warm detection thresholds (WDT) stimuli, respectively. For measuring the cold (CPT) and heat pain thresholds (HPT), the patients pressed the button immediately when the quality of cooling or heating changed into an aching, stinging, or burning sensation (according to the standardized instructions [54]). The temperature range was restricted to 0–50 °C to prevent any skin damage. All four measurements, i.e., thermal detection and pain thresholds for cold and warm/hot stimuli, were repeated five times and then averaged (arithmetic mean) to obtain robust results. The procedure started with testing the thermal detection thresholds for cold and warm stimuli, followed by testing the pain thresholds for cold and hot stimuli accordingly. Reference values of healthy subjects for these thresholds are also available [54].

### 2.7. Statistical Analysis

Statistical analysis was performed with SPSS Version 27.0.1.0 [55] and R Version 4.1.3, including the packages car and ggplot2, using the interface of RStudio Version 2022.2.0.443 [56,57,58,59]. All data were visually inspected for normality using the qqPlot()-function from the car package and were ultimately tested for normality using the Kolmogorov–Smirnov test. Descriptive analysis, testing for normality, and a comparison of the data from measurement sessions T0 and T1 were performed with SPSS. Exploratory data analysis, i.e., a stepwise regression model approach, visual inspection of the data for normality, and data visualization were performed in R. Baseline characteristics and pain characteristics at T0 are reported after descriptive statistical analysis. The psychometric data of the questionnaires were tested for changes that occurred between the measurement sessions (T0 and T1) after the completion of mirror therapy using a paired sample t-test, when the normality of the data could be assumed. Otherwise, the Wilcoxon signed-rank test was used for these comparisons. Similarly, the data of HRV, thermal detection, and pain thresholds were also tested for changes using paired sample t-tests or Wilcoxon signed-rank tests depending on the normality of the data. Post-hoc analysis of the correlations between significant changes in the psychometric and physiological parameters was performed in R using Spearman’s rank correlation coefficient. The data of one patient had to be removed from HRV analysis because the values of absolute power in the low-frequency band were outliers, i.e., over three interquartile ranges above the upper quartile. For the exploratory data analysis of the psychometric and demographic data, a stepwise regression model approach with stepwise selection was applied to identify potential variables that may have influenced the efficacy of the intervention. For this, we used the built-in step() function of R. The stepwise selection method, also called bidirectional elimination, combines a forward and backward selection of the variables, i.e., automatically adding variables to the model at each step that contain significant information according to the Akaike information criterion (AIC) while also removing variables that no longer meet this requirement. For the regression model, we used the reduction of pain intensity (ΔT0 – T1) as a dependent variable and the baseline characteristics and demographic data of the study sample (such as age, sex, BMI, and comorbidities) as well as the baseline values of the DSF scales (in particular, disability, wellbeing, depression, anxiety, and stress scale) as possible predictors that the stepwise selection method could choose. The alpha level of significance was set to *p* < 0.05 for all statistical analyses.

## 3. Results

### 3.1. Psychometric Parameters

#### 3.1.1. Baseline Measurements at T0

The DSF included an assessment of pain characteristics at the first measurement session (T0) to allow for a description of the history of pain symptoms and their qualities in the study sample. The baseline characteristics and demographic data of the study sample are shown in Table 1.

Concerning the pain symptom duration of the fifteen patients, four patients (26.67%) reported having pain symptoms for more than five years, six patients (40%) had been experiencing their pain symptoms for two to five years, three patients (20%) reported a duration of one to two years, and two patients (13.3%) reported a duration of their pain symptoms for six months to a year.

The patients were also questioned about their pain profile, i.e., if the pain was more experienced in the form of attacks or in a continuous manner, and if there were pain-free intervals or fluctuations in severity. Four patients (26.7%) reported having primarily pain attacks with pain persisting in between, one patient (6.67%) was also having pain attacks, but with pain-free intervals in between the attacks. Six patients (40%) reported having mainly continuous pain with severe fluctuations, and four patients (26.7%) were also having continuous pain but with only slight fluctuations.

The main pain complaints of the patients were primarily localized in the back (six patients, 40%) and the upper extremities (five patients, 33.3%). The lower extremities (two patients, 33.3%), thorax (one patient, 6.7%), and head (one patient, 6.7%) were less often reported. As required by the inclusion criteria, the stated pain complaints were lateralized, i.e., they only appeared or were predominant on one side of the body.

#### 3.1.2. DSF Comparison of T0 and T1

**Figure 2:**
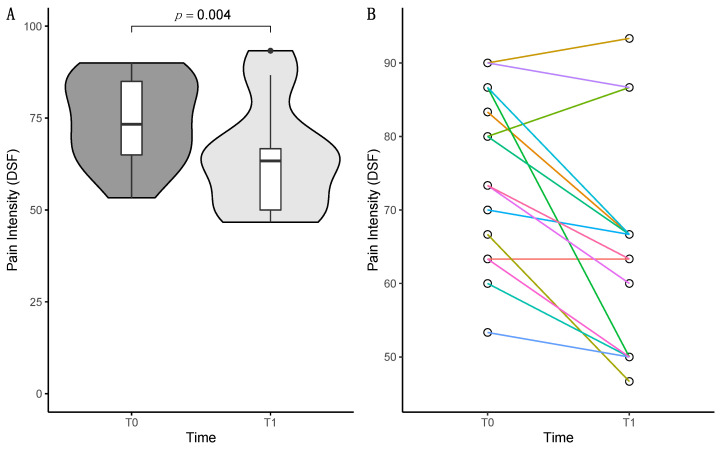
(**A**) Violin plot with nested box plot of pain intensity as measured with the DSF. Pain intensity was significantly reduced comparing values of T0 (dark grey) and T1 (light grey); (**B**) connected scatterplot of pain intensity data as measured with the DSF, each color represents the data of a corresponding patient.

A comparison of the DSF pain intensity scale before (T0) and after (T1) mirror therapy showed a significant reduction (*z* = −2.878, *p* = 0.004, Table 2 and Figure 2). Individual analysis of the NRS items of the pain intensity scale revealed that the average pain in the last four weeks was reduced through mirror therapy (*t*(14) = 3.850, *p* = 0.002, Table 2). The reduction of current pain (z = −1.897, *p* = 0.058, Table 2) and greatest pain in the last four weeks (z = −1.812, *p* = 0.070, Table 2) failed to be statistically significant. The improvement in disability failed to reach statistical significance as well (*z* = −1.121, *p* = 0.262, Table 2). Analysis of the subscales for wellbeing, depression, anxiety, and stress showed no significant differences (all *p*s > 0.05, Table 2).

#### 3.1.3. Exploratory Data Analysis of the Reduction in Pain Intensity

The stepwise regression method used to identify the variables that predicted the improvement of pain symptoms yielded an overall significant regression model (*F*(3,11) = 8.638, *p* = 0.003, *R*^2^ = 0.702), with the following significant predictors: comorbidity depressive disorder (β = 16.65, *SE* = 4.53, t(11) = 3.674, *p* = 0.004), disability (DSF) (β = −2.61, *SE* = 1.05, *t*(11) = −2.476, *p* = 0.031), and depression (DASS) (β = −0.92, *SE* = 0.34, *t*(11) = −2.710, *p* = 0.020), indicating that patients with depression as a comorbidity had a greater reduction in pain intensity compared to patients with somatoform pain disorder without depression. The model also suggests that patients with lower levels of disability and depressive symptoms at baseline may have been a predictor for a greater reduction in pain intensity. A visual inspection of the model residuals with a Q-Q plot indicated no violation of the assumption of normal distribution.

### 3.2. Physiological and Psychophysical Parameters

#### 3.2.1. HRV Comparison of T0 and T1

Analysis of the parameters of HRV showed a decrease in the absolute power in the low-frequency band after the completion of mirror therapy (*t*(13) = 2.536, *p* = 0.025, Table 3 and Supplementary Figure S1A). However, this effect vanished when comparing the normalized power values of the low-frequency band (*t*(13) = 0.169, *p* = 0.868, Table 3). No other statistically significant changes in the remaining HRV parameters were detected (all *p*s > 0.05; Supplementary Figure S1B and Table 3).

#### 3.2.2. Thermal Detection and Pain Threshold Comparison of T0 and T1

Examination of the thermal detection and pain thresholds showed an increased value for CPT when comparing the thresholds before (T0) and after (T1) the therapy regimen (*z* = −2.040, *p* = 0.041, Table 3 and Supplementary Figure S2A), i.e., the tolerance of the subjects for cold stimuli decreased, that is, they were more sensitive to these stimuli. A comparison of the remaining thresholds yielded no statistically significant differences (all *p*s > 0.05; Supplementary Figure S2B and Table 3).

## 4. Discussion

In this study, the potential efficacy of a four-week mirror therapy program for patients with a chronic somatoform pain disorder was examined for the first time. It was evaluated if and to what extent pain intensity may be reduced and if it was associated physiological and psychophysical parameters, i.e., HRV and sensitivity to painful stimuli were also altered by the intervention. The findings of this study are: (i) mirror therapy led to significantly reduced pain levels after four weeks of intervention, where, specifically, the average pain intensity in patients was reduced; (ii) the absolute power but not the normalized power of HRV in the low-level band was significantly decreased after the performance of mirror therapy; (iii) pain sensitivity to only cold painful stimuli increased after four weeks of mirror therapy; and (iv) patients with comorbid depressive disorder particularly benefitted, regarding pain reduction, from the mirror therapy.

The overall pain intensity, as measured by the respective DSF scale, was significantly reduced through the therapy program. Our results are in line with studies on CRPS, which demonstrated the efficacy of the mirror beyond phantom limb pain [21,60]. The differentiated analysis of the individual items that make up the pain intensity scale revealed that the average pain intensity of the last four weeks was reduced, but not the current or greatest pain intensity. However, there seems to have been no effect of the intervention on the disability or wellbeing of the patients or on non-pain-related symptoms, such as anxiety and depression. Our exploratory data analysis suggests that patients with a comorbidity of a depressive disorder particularly benefitted from mirror therapy, thereby identifying a subgroup of patients where mirror therapy may be evaluated and investigated first in subsequent studies. One has to note that the degrees of disability and depressive symptoms were negative predictors of the efficacy of mirror therapy in pain reduction, i.e., the more severe the depressive symptoms and disabilities were, the smaller the improvements regarding pain. Overall, the results suggest that patients with an additional diagnosis of depression may benefit especially from the intervention, but that this effect may be attenuated by the degree of depressive symptoms and disability.

We also found that the cold pain threshold values were significantly increased after mirror therapy compared to the thresholds at baseline. This suggests that patients in this study became more sensitive to painful cold stimuli after completion of the therapy program. Achenbach et al. [61] conducted qualitative sensory testing in patients with multisomatoform pain disorders and compared this patient group to healthy controls. At baseline, our study sample had similar cold pain thresholds as the control group in the aforementioned study. After the intervention, our sample showed increased thresholds, similar to the patients with multisomatoform chronic pain disorders [61] and patients with chronic localized or widespread pain [62]. These findings were not expected, especially in light of our finding that the average pain intensity of the patients in our study was reduced after the mirror therapy intervention. One would rather assume that the increased sensitivity to painful thermal stimuli, as found in various chronic pain disorders [36,37,61,62] would also decrease after an intervention if the pain symptoms are improved by the intervention. The heat pain thresholds in our study sample were not altered by our intervention. This is in line with the findings of Achenbach et al. [61], who demonstrated that patients with multisomatoform pain disorders exhibited similar heat pain thresholds as healthy controls. These differences in cold and heat pain thresholds may be explained by the assumption that increased sensitivity to painful cold stimuli, i.e., cold hyperalgesia, is facilitated by the mechanism of central sensitization, whereas heat hyperalgesia may be mainly influenced by peripheral sensitization [63]. Our findings, therefore, rather indicate that in somatoform pain disorders, central and not peripheral sensitization is involved. Further studies could, therefore, specifically look at whether mirror therapy has a differential effect on thermal pain thresholds or the processes of central and peripheral sensitization, apart from a reduction in overall pain symptoms. Looking at the thermal detection thresholds, these were not altered in our study. Comparing our sample with the data of Achenbach et al. [61], we found values more similar to those of their control group. However, one must keep in mind that these metrics may not be best suited for measuring differences in this patient population, as, for example, Gerhardt et al. [62] found no differences when comparing the cold detection thresholds of patients with chronic pain disorders with those of healthy controls. Overall, when looking at the reference data for thermal pain thresholds [54], one sees that the confidence intervals for the thermal pain thresholds turned out to be so large that the data from our sample, whether measured before or after our intervention, still fell within the normal range. When interpreting our results of the thermal thresholds, however, it is important to note that we omitted a familiarization procedure from the test in order not to exceed the time frame of the first measurement session of our study. This procedure familiarized the patient with the applied stimuli and allowed for practice with the response format as well. This can partially eliminate sources of measurement errors, such as mistakenly pressing the response button or not fully utilizing the temperature spectrum due to stopping the measurement too early. Therefore, this may have had an impact on the accuracy, and especially the variance, of our data.

Our final finding was the reduction of absolute power in the low-frequency band of HRV. Previous research has shown that patients with somatic symptom disorders and functional somatic syndromes can show reduced HRV [33], and HRV is also able to predict the therapy outcomes of several treatment approaches for somatoform disorders [64]. Studies have also suggested that HRV is connected to induced pain perception and intensity [65]. Induced pain, therefore, increases power in the low-frequency band [66,67]. However, the results of HRV changes in chronic pain conditions and somatoform disorders are often very heterogeneous [31,33], with some studies showing no differences regarding high-frequency HRV or even increases in low-frequency HRV [68].

Because the patients in our study showed a reduction in pain intensity and the absolute power of low-frequency HRV, our results could therefore be consistent with the assumption of a positive relationship between pain intensity and low-frequency HRV (post-hoc correlational analysis of the changes found in low-frequency HRV and pain intensity yielded, however, no statistically significant correlation: *r*_s_(12) = −0.48, *p* = 0.084). As HRV is thought to have a normal or healthy range [69], in comparing the HRV of patients in this study with the normal values of healthy adults [52,70], the change in the low-frequency power, i.e., values decreasing towards those of healthy individuals, could also be conceptualized as a renormalization of HRV. Yet, one should keep in mind that not all interventional studies on chronic pain conditions have shown changes in HRV [71], and the reduction in absolute power in the present study vanished when analyzing the normalized power of the low-frequency band. Using normalized units is preferable, as the results are thought to be more robust and methodologically reliable [41]. However, the use of normalized units may lead to an underestimation of the changes in power [51,72]. Additionally, it must be noted that we only found changes in the low-frequency but not the high-frequency HRV. As parasympathetically mediated high-frequency HRV is the most robustly altered HRV metric in somatoform (pain) disorders [33], we would have expected to see the high-frequency HRV also be altered, reflecting the reduction in the pain intensity of the patients. Although the separation of SNS and PNS influences on low- and high-frequency HRV has also been debated critically [41,52], our results may indicate that mirror therapy has more of an influence on the sympathetic and less on the parasympathetic component of HRV. This also may mean, however, that the pain-reducing effects of mirror therapy would have to be even more pronounced before being reflected in the metric of high-frequency HRV. To this end, it must be said that the data of our patients sometimes show a very large variance in HRV metrics. As our patients did not have any restrictions imposed on their activities before the measurements (e.g., regarding coffee or nicotine intake), due to practicability reasons, our results also have to be considered in view of these possible reasons leading to higher variability. Therefore, one cannot exclude the possibility that the changes found in HRV in our study were also due to natural fluctuations, possibly also caused by, for example, sporting activity or the consumption of substances, such as caffeine, that have an influence on HRV (e.g., [41,73,74]).

To our knowledge, this study is the first of its kind to investigate the effect of mirror therapy as a novel therapeutic approach in patients with chronic somatoform pain disorders. New approaches are especially needed, as these patients often remain symptomatic even after extensive diagnostic and therapeutic interventions. However, several open questions and limitations remain with our study. As our study featured no control group, a clear causative attribution of the reduction in symptoms to our intervention cannot be made. Comparing mirror therapy to a treatment group with more unspecific training activity could further elucidate the specific mechanisms of mirror therapy that underlie its efficacy. Patients in this study did not exclusively report pain in the extremities. While mirror therapy is an intervention designed to facilitate improvement in pain or disabilities in the extremities, a possible mechanism of action in lateralized pain not localized in the extremities, such as in the shoulders, lower back, or hips, needs to be investigated, warranting further studies involving and comparing different groups of patients with chronic pain in various locations. Possible mechanisms of action may be an alleviation of symptoms via increased body awareness or states of mindfulness that occur during mirror therapy or unspecific effects of physical activity, which have been shown to reduce symptoms in patients with chronic pain [75,76]. Another limitation of our study is also the fact that we were not able to precisely monitor the adherence of the patients to the intervention. While the patients affirmed that they complied with the program, objective confirmation of this would be desirable in future studies. Moreover, as the patients were recruited consecutively, we did not control for the pain state of the participating patients, e.g., if they were pain-free or in a painful state (as one patient in our study had a pain profile with the former course). This may have influenced our measurements and the efficacy of our intervention. Yet, all participating patients needed to indicate that they felt able and well to participate in the study and could have discontinued their participation in case they felt unwell or had other particular complaints.

Additionally, as our study also consisted of a rather small sample size and did not include follow-up measurements, the robustness and long-term effects of our findings remain unclear. Therefore, randomized controlled studies with larger cohorts are mandatory to further investigate the effects of mirror therapy in somatoform pain disorders. Lastly, as mirror therapy is relatively cost-efficient and low in side effects, it poses a promising addition to the existing therapy options in the multimodal treatment of patients with chronic somatoform pain. Since the intervention would be relatively inexpensive to implement, it would likely fit fairly seamlessly into treatments that already focus on the patient’s body, such as biofeedback therapy and mindfulness exercises.

## 5. Conclusions

In summary, our pilot study provides the first evidence that mirror therapy may be an efficacious additional therapy approach within a multimodal pain therapy program for patients with chronic somatoform pain disorders. However, several limitations of this study remain that restrain its conclusiveness regarding the potential pain-reducing effects of mirror therapy in these patients.

## Figures and Tables

**Table 1:** Baseline characteristics of *n* = 15 participants.

Parameter	Distribution: *n* (%), Median (Q1–Q3)
Female	9 (60)
Age (years)	39 (28–55)
BMI (kg/m^2^)	28.5 (26.00–29.25)
Children (yes)	9 (60)
Marriage (yes)	9 (60)
*Diagnosed Comorbidities*	
Depressive disorder	9 (60)
Anxiety disorder	4 (26.7)
*Medication at Baseline*	
Antidepressant	5 (33.3)
Antipsychotics	1 (6.7)
Anticonvulsant drugs	4 (26.7)

Note: BMI, body mass index.

**Table 2:** Comparison of psychometric parameters before and after mirror therapy. Value ranges of each questionnaire are shown in square brackets.

Questionnaire	T0: *M* (*SD*)	T1: *M* (*SD*)	Test Value	*p* Value
DSF				
Pain intensity [0–100]	74.67 (11.67)	64.44 (14.67)	*z* = −2.878	0.004 *
Current pain [0–10]	6.33 (1.84)	5.73 (2.05)	*z* = −1.897	0.058
Average pain [0–10]	7.00 (1.20)	5.80 (1.74)	*t*(14) = 3.850	0.002 *
Greatest pain [0–10]	9.07 (1.39)	7.80 (2.46)	*z* = −1.812	0.070
Disability [0–6]	4.40 (1.77)	3.87 (2.03)	*z* = −1.121	0.262
MFHW				
Wellbeing [0–35]	12.27 (8.84)	14.27 (9.53)	*t*(14) = −1.651	121
DASS				
Depression [0–21]	15.07 (6.75)	15.00 (5.92)	*t*(14) = 0.066	948
Anxiety [0–21]	13.07 (6.03)	12.67 (5.62)	*z* = −0.448	654
Stress [0–21]	15.60 (5.55)	14.80 (5.72)	*t*(14) = 0.939	364

Note: DSF, German Pain Questionnaire; average and greatest pain refer to the period of the last four weeks; MFHW, Marburg Questionnaire on Habitual Health Findings; DASS, Depression, Anxiety, and Stress Scale; * indicates statistically significant differences.

**Table 3:** Comparison of physiological parameters before and after mirror therapy.

Measure	T0: *M* (*SD*)	T1: *M* (*SD*)	Test Value	*p* Value
HRV				
Mean RR (ms)	804.14 (149.62)	743.59 (138.23)	*t*(13) = 1.83	0.091
RMSSD (ms)	23.94 (10.71)	20.57 (11.64)	*t*(13) = 0.859	0.406
LF absolute (ms^2^)	659.64 (473.93)	372.46 (239.18)	*t*(13) = 2.536	0.025 *
HF absolute (ms^2^)	230.07 (155.95)	213.93 (202.75)	*z* = −0.722	0.470
LF normalized (nu)	72.18 (12.05)	71.35 (17.31)	*t*(13) = 0.176	0.863
HF normalized (nu)	27.78 (12.04)	28.57 (17.20)	*t*(13) = −0.169	0.868
LF/HF ratio	3.19 (1.63)	4.35 (5.19)	*z* = −1.036	0.300
Thermal detection thresholds (in °C)				
CDT	29.65 (2.56)	30.44 (1.63)	*z* = −1.070	0.285
WDT	34.71 (1.84)	34.81 (2.15)	*t*(14) = −0.164	0.872
Pain thresholds (in °C)				
CPT	12.33 (11.30)	15.37 (11.94)	*z* = −2.040	0.041 *
HPT	43.25 (4.56)	43.46 (4.32)	*z* = −0.995	0.320

Note: CDT, cold detection threshold; WDT, warm detection threshold; CPT, cold pain threshold; HPT, heat pain threshold; RR, interval between successive heartbeats; RMSSD, root mean square of successive differences; LF, low frequency; HF, high frequency; * indicates statistically significant differences.

## Data Availability

The datasets generated during and/or analyzed during the current study are available from the corresponding author upon reasonable request.

## Supplementary Materials

The following supporting information can be downloaded at: https://www.mdpi.com/article/10.3390/bs13050432/s1, Figure S1: Violin plots of HRV with nested box plots at T0 (dark grey) and T1 (light grey); Figure S2: Violin plots of pain thresholds with nested box plots at T0 (dark grey) and T1 (light grey).
