# Supplementary material for: Mirror Therapy in Patients with Somatoform Pain Disorders—A Pilot Study
Source: Behav Sci (Basel). 2023 May 20;13(5):432. doi: 10.3390/bs13050432 (PMC10215185; doi:10.3390/bs13050432)
Supplement: Supplementary file 1 [file behavsci-13-00432-s001.zip › behavsci-2342708-supplementary.pdf]

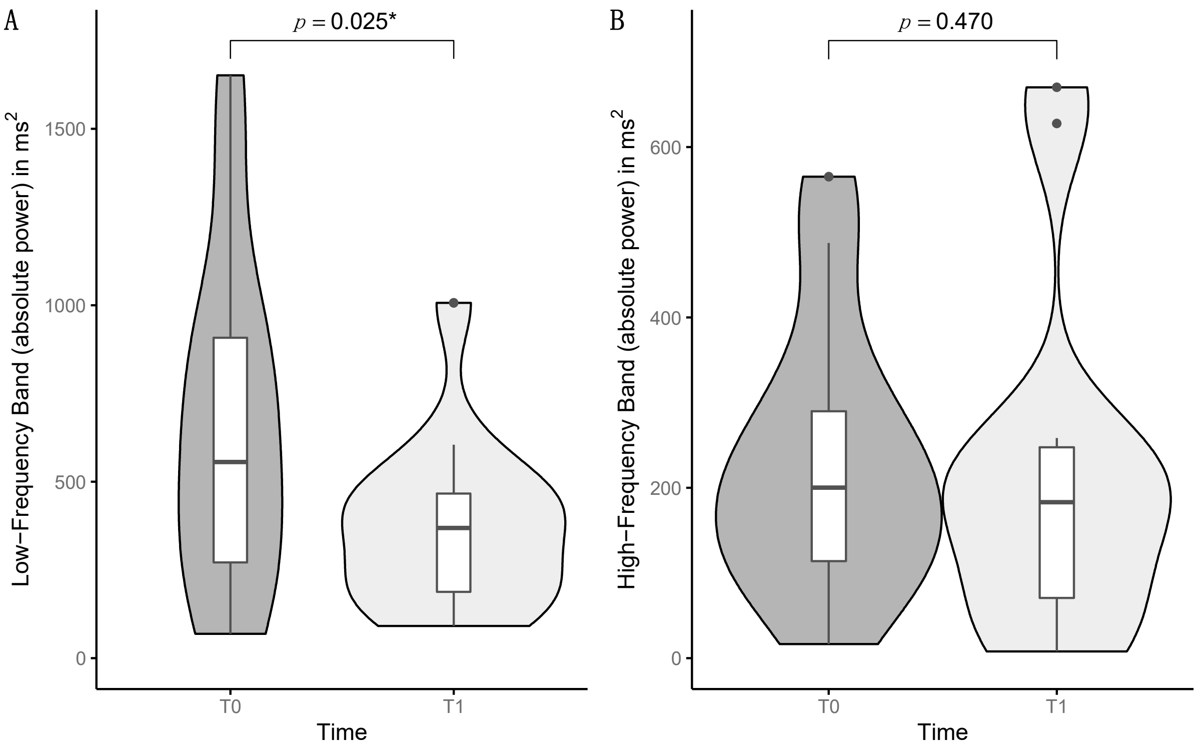

Figure S1. Violin plots of HRV with nested box plots at T0 (dark grey) and T1 (light grey). (**A**) HRV in the low-frequency band (absolute power) was significantly decreased; (**B**) HRV in the high-frequency band (absolute power) was not significantly altered.

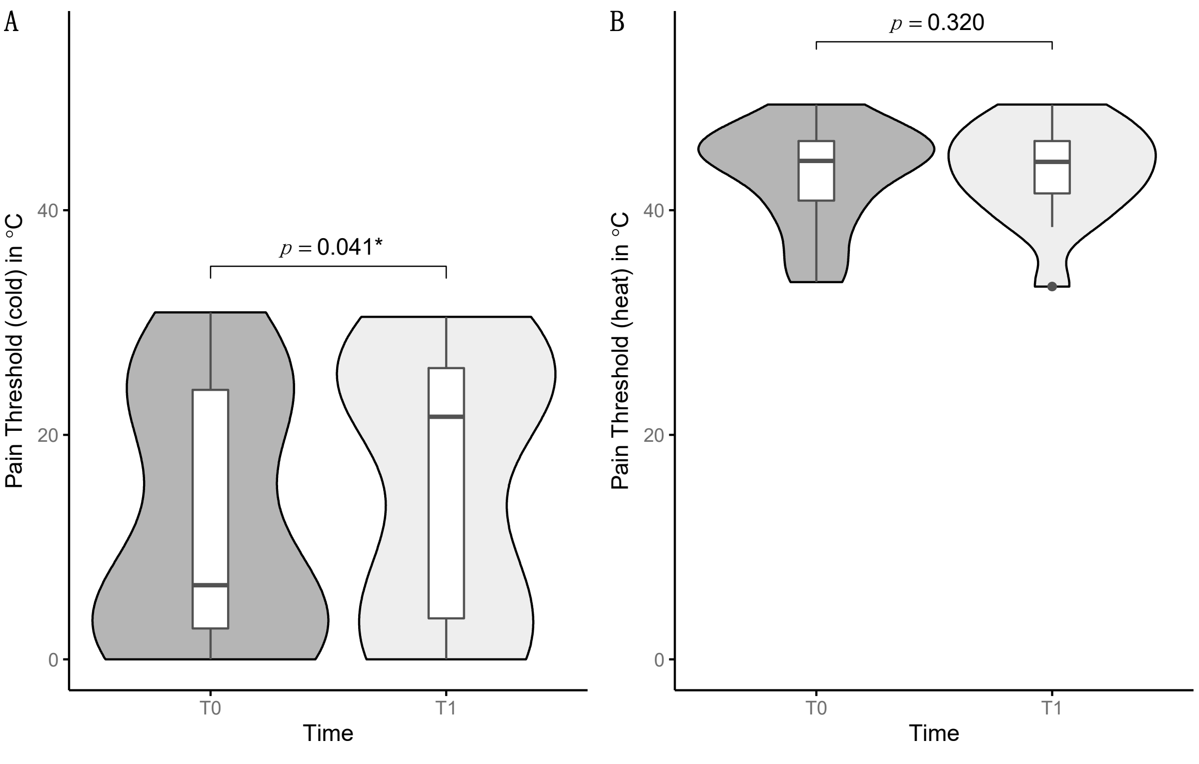

Figure S2. Violin plots of pain thresholds with nested box plots at T0 (dark grey) and T1 (light grey). (**A**) Pain thresholds for cold stimuli were significantly decreased, i.e. subjects were more sensitive to cold stimuli; (**B**) Pain thresholds for heat stimuli did not show any significant changes.
